# Structural insights into Phospholipase Cε activity and regulation

**DOI:** 10.1016/j.jbc.2026.111289

**Published:** 2026-02-16

**Authors:** Ketaki A. Mahurkar, Stephanie L. Barrios, Faith D. McCauley, Angeline M. Lyon

**Affiliations:** 1 James Tarpo Jr. and Margaret Tarpo Department of Chemistry, Purdue University, West Lafayette, Indiana, USA; 2 Department of Biological Sciences, Purdue University, West Lafayette, Indiana, USA

**Keywords:** G protein, heterotrimeric G protein, phospholipase C, PIP_2_, protein structure, phosphatidylinositol signaling, phosphatidylinositol, second messenger, small GTPase

## Abstract

Phospholipase Cε (PLCε) is a complex, multifunctional enzyme that responds to and integrates signals from G protein-coupled receptors and receptor tyrosine kinases through the direct binding of G proteins. These activators translocate PLCε to the cytoplasmic leaflets of the plasma and perinuclear membranes where the lipase hydrolyzes phosphatidylinositol lipids to produce inositol polyphosphates and diacylglycerol. These second messengers increase intracellular Ca^2+^ and/or activate protein kinase C, respectively, stimulating numerous pathways. Recent studies have broadened our understanding of this enzyme, revealing roles for PLCε in Ca^2+^-induced Ca^2+^-release processes in the kidneys and pancreas, as well as in cancer. These are complemented by structural studies that provide more complete insights into its basal and G protein-bound conformations. Here, we summarize and discuss these advances in understanding the regulation and roles of PLCε in normal and pathological contexts.

Phospholipase Cs (PLCs) are peripheral membrane proteins that hydrolyze phosphatidylinositol (PI) lipids at the cytoplasmic leaflets of membranes, generating second messengers that activate downstream signaling pathways (1). At the plasma membrane, PLCε hydrolyzes phosphatidylinositol-4,5-bisphosphate (PIP_2_) (2), generating inositol-1,4,5-triphosphate (IP_3_) and diacylglycerol (DAG). These molecules increase intracellular Ca^2+^ and activate protein kinase C (PKC). At the perinuclear membrane and the Golgi, PLCε cleaves phosphatidylinositol-4-phosphate (PI4P), generating inositol-1,4-phosphate (IP_2_) and DAG (3). DAG activates PKC, and together they activate protein kinase D.

Two splice variants of PLCε, PLCε1a and PLCε1b, have been reported (4). PLCε1a is widely expressed, particularly within the skeletal muscle, thymus, testes, placenta, lung, and spleen (4). PLCε1b is more abundant in the placenta, lung, and spleen, but is not detected in the other tissues (4). The isoforms are encoded by a single gene in humans and differ in the length of exon 1 (4). This results in the PLCε1a protein having an additional 308 residues at its N-terminus relative to PLCε1b (4). Both variants are predicted to share the conserved domains observed in other PLC enzymes, including a pleckstrin homology (PH) domain, four EF hand repeats, a catalytic triose phosphate isomerase (TIM) barrel split into X and Y halves by an X–Y linker, and a C2 domain (5, 6, 7, 8). The PLCε enzymes are unique in that they contain a cell division and cycle 25 (CDC25) homology domain at their N-terminus, an insertion termed the Y-box within the Y half of the TIM barrel, and two C-terminal Ras association (RA) domains, RA1 and RA2 (5, 9, 10) (Fig. 1*A*). The majority of studies have focused on PLCε1a, referred to as PLCε in the literature and in this review.

**Figure 1:**
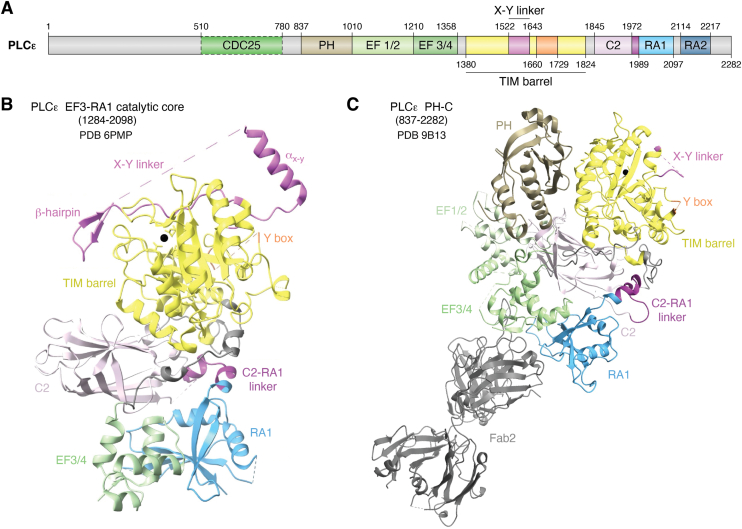
**DomainOrganization of Phospholipase Cε**. *A*, Domain diagram of *Rattus norvegicus* phospholipase Cε. Numbers above correspond to experimentally determined domain boundaries. Domain boundaries (*dashed lines*) for the CDC25 domain are based on AlphaFold3 predictions (34). The X-Y linker is shown in *pink*, the Y-box in *orange* and the C2-RA1 linker in *dark purple*. *B*, Crystal structure of the PLCε EF3-RA1 catalytic core (residues 1284–2098, PDB ID: 6PMP) colored as in 1A. The catalytic Ca^2+^ is shown as a *black sphere*, and disordered regions of the X–Y linker and Y-box are shown in dashed lines. *C*, Cryo-EM structure of PLCε PH-C (residues 837–2282), colored as in 1A, bound to an antigen binding fragment, Fab2, (*gray*) (PDB ID: 9B13). Disordered regions are shown as *dashed lines*. CDC25, cell division and cycle 25; PLCε, phospholipase Cε.

In resting cells, the lipase is maintained in a low activity state in the cytoplasm, but in response to stimulation of cell surface receptors, namely G protein coupled receptors (GPCRs) and receptor tyrosine kinases, PLCε is translocated and activated at cytoplasmic leaflets of membranes through direct binding of small G proteins, including Rap1A, RhoA, and Ras (11, 12), and the heterotrimeric G subunit Gβγ (13). In this review, we highlight the physiological roles of PLCε and summarize recent structural and functional studies into the molecular mechanisms of PLCε regulation.

## Structures of PLCε

Full-length PLCε has been recombinantly expressed and purified (5), but sufficient quantities for structural and biochemical studies have not yet been obtained. Most biochemical studies and all structural studies to date have used individual domains or catalytically active fragments of the protein. The first structural insights into PLCε were solution structures of its C-terminal RA domains, alone and in tandem, determined by nuclear magnetic resonance (9). Subsequent studies relied on N-terminal truncations of the lipase, beginning at its PH domain or EF hands, and extended through the C2, RA1, or RA2 domains (6, 8, 14, 15, 16). Small angle X-ray scattering and negative stain EM revealed the overall architecture of these truncations and their conformational heterogeneity in solution (8, 16). This work led to the first high-resolution structure of the PLCε catalytic core, which spans EF hands 3/4 through the RA1 domain (6) (PDB ID: 6PMP) (Fig. 1*B*). This structure provided insight into the architecture of the minimal catalytically active fragment of the lipase, revealed subfamily-specific ordered regions within the autoinhibitory X–Y linker, and showed the RA1 domain is an integral part of the catalytic core of PLCε (6).

The largest variant of PLCε that has been structurally characterized is PLCε PH-C (residues 837–2282), which lacks the N-terminal 836 residues. This variant has robust basal activity in cell-based and liposome-based activity assays, and is activated by small GTPases (6, 8, 16). Efforts to determine high-resolution structures of this variant were initially hindered by conformationally heterogeneity of the PH domain and EF hands 1/2 (EF1/2), which do not stably interact with the catalytic core (8). Structures of this variant, bound to an antigen binding fragment (Fab) (PDB ID: 9B13) (7) (Fig. 1*C*) or to one of its activators, RhoA•GTP (PDB ID: 9AX5), (17) were recently determined using cryo-EM single particle analysis. These structures help define the basal and G protein-bound states of the lipase and allowed experimental determination of the N-terminal PH and EF hand domains, the latter of which features an unusual architecture that is essential for RhoA-dependent activation.

**Figure 2:**
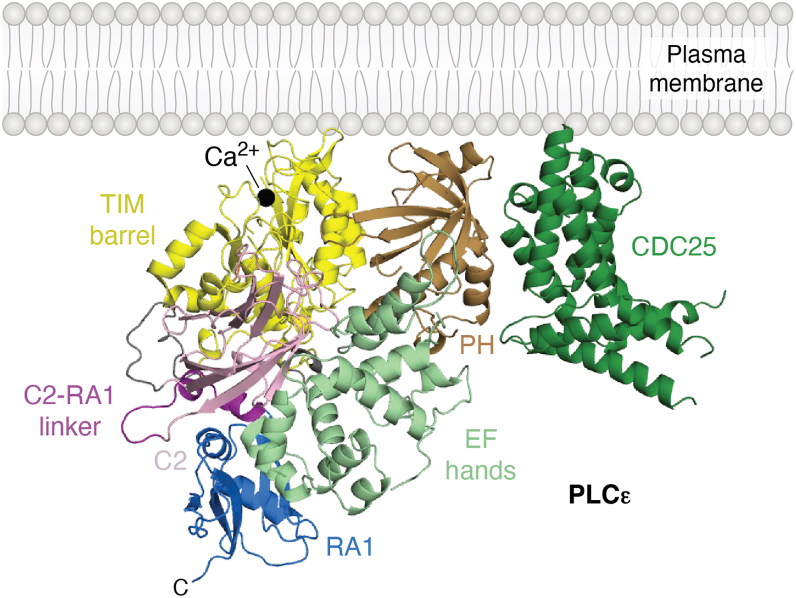
**The CDC25 and PH domains form a putative membrane binding interface**. The AlphaFold model of PLCε CDC25-RA1 reveals an interface between the CDC25 domain (*green*) and the PH domain. This forms an extended basic and hydrophobic surface in the same plane as the active site in the TIM barrel, indicated by the catalytic Ca^2+^ (*black sphere*). The X–Y linker is not shown for clarity. PLCε is colored as in Figure 1*A*. CDC25, cell division and cycle 25; PLCε, phospholipase Cε.

The PLCε PH domain contributes to lipase activity, most likely through membrane association, as N-terminal truncations or deletion of this domain decrease activity and impair binding to PIP_2_-containing liposomes (6, 8). It does not contribute to stability, indicating the domain interacts transiently with the catalytic core (8). This is supported by the cryo-EM reconstructions, in which the PH domain is adjacent to the TIM barrel, but compared to this interface in other PLC structures, fewer interdomain interactions are observed (2, 13, 18, 19, 20). This is reminiscent of the PLCδ PH domain, which tethers the rest of the catalytic core to the membrane. However, unlike PLCδ, the residues required for PIP_2_ binding are not conserved in PLCε. Instead, the domain most likely interacts non-specifically with the membrane, as has been reported for the PLCβ subfamily (21, 22). The surface of the PH domain that lies in the same plane as the active site in the TIM barrel features solvent-exposed basic and hydrophobic residues, which are consistent with nonspecific binding. In AlphaFold3 models, this surface is extended by the CDC25 domain, which is also predicted to interact with the PH domain. If true, this would allow the CDC25, PH, and TIM barrel domains to simultaneously bind a shared membrane surface (7) (Fig. 2).

The Fab–PLCε PH-C reconstruction confirmed the lipase contains four tandem EF hand repeats, and, like most other PLCs, the EF hands lack the canonical Ca^2+^ binding residues (7). An early bioinformatics study attempted to map its secondary structure, but this was complicated by low sequence conservation of this region (19% identity across PLCs) (13). The architecture of the PLCε EF hands are most similar to PLCβ, wherein the EF1/2 and EF3/4 lobes each contain four helices (23, 24). Interestingly, the PLCε EF1/2 lobe is structurally completed by an approximately eighty residue insertion in EF3/4. This region folds back onto EF1/2, contributing the fourth helix of the bundle before re-entering the EF3/4 subdomain (7). This helix, referred to as E2α′ based on the architecture of PLCβ, is the binding site for the RhoA GTPase, and is described in subsequent sections (17). While EF1/2 is not required for catalytic activity, the EF3/4 subdomain is, as it makes extensive interactions with the C2 and RA1 domains that stabilize the catalytic core (6) (Fig. 1*C*).

**Figure 3:**
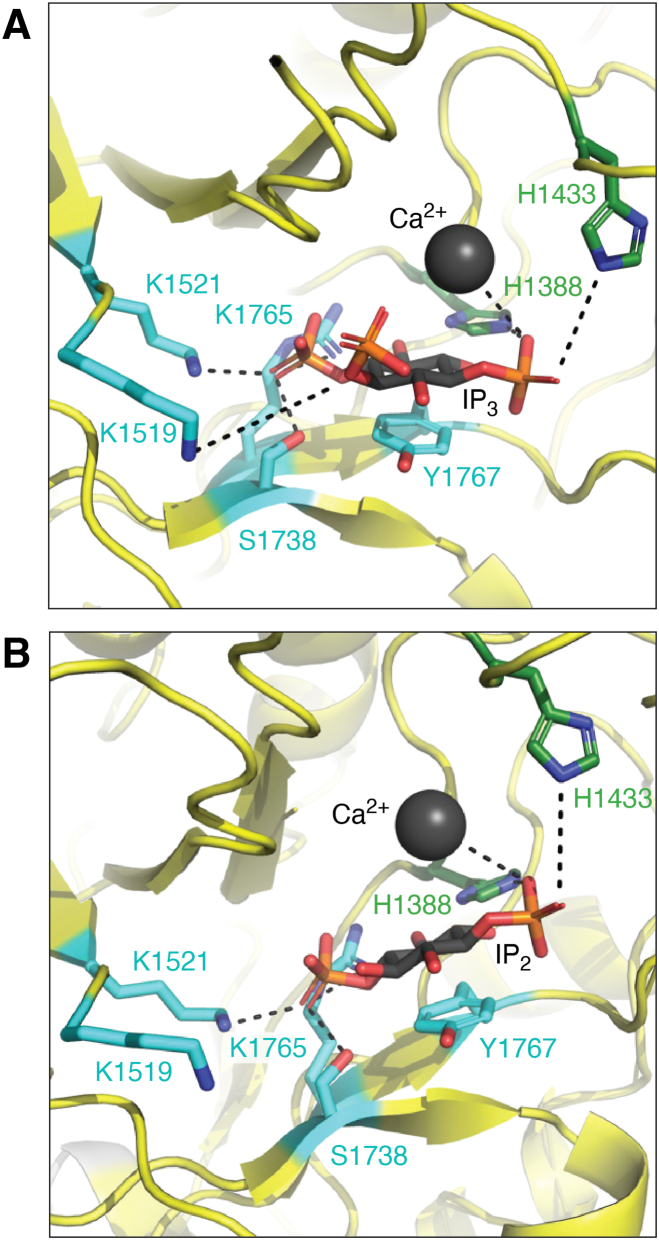
**PLCε hydrolyzes PIP_2_ and PI4P**. *A*, The active site of PLCε (PDB ID: 9B13) modeled with IP_3_, a product of PIP_2_ hydrolysis. The 1-phosphate group is coordinated by catalytic Ca^2+^ (*black*) and H1388 and H1433. Residues K1519, S1738, K1765 coordinate the 4-phosphate group, while K1521 coordinates the 5-phosphate group. *B*, The PLCε active site modeled with IP_2_, a product of PI4P hydrolysis. Because PI4P lacks the 5-phosphate group, K1521 does not participate in substrate binding (2). PLCε, phospholipase Cε; IP_3,_ inositol-1,4,5-triphosphate; PIP_2_, phosphatidylinositol-4,5-bisphosphate.

The TIM barrel is highly conserved across PLCs; all are Ca^2+^-dependent enzymes and share the same active site residues (2, 24, 25) (Fig. 3). Adjacent to the active site is a highly conserved, solvent-exposed hydrophobic loop, referred to as the hydrophobic ridge. Structural and functional studies of product-bound PLCδ complexes (2) and PLCβ3 (19) suggest this ridge inserts into the lipid bilayer, anchoring the active site at the membrane and allowing PIP_2_, or other phosphatidylinositol phosphate (PIP) substrates, to bind the active site (2, 26). The mechanism of PIP hydrolysis was proposed based on studies of PLCδ. Briefly, Ca^2+^ coordinates the phosphate groups of the substrate. H311 (*Rattus norvegicus* PLCε H1388) deprotonates the 2-hydroxyl group of the PIP head group, forming a cyclic phosphodiester intermediate, and releasing DAG (2). In the second step, the cyclic intermediate is hydrolyzed by H356 (*R*. *norvegicus* PLCε H1433), releasing IP_3_ or other inositol phosphate species (2, 26) (Fig. 3).

The PLCε TIM barrel contains two regulatory insertions, the X–Y linker and the Y-box. In PLCε, PLCβ, and PLCδ enzymes, the X–Y linker inhibits activity by blocking the active site until it is displaced by interfacial activation at the membrane. The crystal structure of the PLCε EF3-RA1 catalytic core revealed an amphipathic helix (α_X–Y_) at the N-terminus of the X–Y linker (Fig. 1*B*), but the entire region is disordered in cryo-EM reconstructions, consistent with it being conformationally dynamic in solution. The mechanism by which the X–Y linker is proposed to regulate activity is described in the following section. In contrast, little is known about the Y-box, which is adjacent to the X–Y linker. Initially, this element was proposed to essential for regulation by the RhoA GTPase, and potentially contribute to binding the G protein (14). More recent work has shown that it is necessary for enzymatic activity, but its role is not known (17).

The C2 domain makes extensive interactions with the TIM barrel, EF3/4 hands, and in PLCε, the RA1 domain (Fig. 1, *B* and *C*) (2, 6, 7, 15, 17, 18, 19, 27, 28, 29, 30). While the C2 domain in PLCδ has been proposed to bind Ca^2+^ (31, 32) and contribute to membrane association, these residues are not conserved in PLCε. The C2 domain likely functions in part as a scaffolding domain. Indeed, the C2-RA1 linker binds to a hydrophobic pocket at the TIM barrel-C2 interface. Mutation of the linker or its binding site increases basal activity, suggesting it may have a regulatory role (6). PLCβ activity is also regulated by a helix that binds to the cleft between the TIM barrel and C2 domains (27), indicating this interface may be an important regulatory surface across PLCs.

PLCε is the only member of the PLC family that contains RA domains. While they are structurally homologous to RA domains in other proteins, the PLCε RA1 domain does not bind activated G proteins (5, 9). Instead, its predicted G protein binding surface interacts extensively with the EF3/4 hands and C2 domain (6) (Fig. 1, *B* and *C*). Mutations that disrupt the EF3/4-RA1 or C2-RA1 interfaces decrease stability and basal activity of the lipase (6). The RA1 domain has also been shown to bind the muscle-specific A kinase anchoring protein (mAKAP) at the perinuclear membrane in cardiomyocytes. PLCε variants lacking the RA1 and/or RA2 domains were impaired in localization, indicating they are necessary for scaffolding (33). Binding to mAKAP may disrupt the binding of the C2-RA1 linker to the TIM barrel-C2 interface, providing another avenue to increase lipase activity, but this remains to be determined.

Lastly, the C-terminal RA2 domain is present in both reconstructions of PLCε PH-C complexes, but no density for it was observed, consistent with its being flexibly connected (7, 9, 16, 17). Whether the RA2 domain contributes to basal activity and/or autoregulation is unclear, as its deletion variably impact basal activity (5, 9, 16). The best characterized role of this domain is as the primary binding site for the Ras and Rap1A GTPases, both of which robustly activate PLCε and are described in later sections (5, 9, 11, 16).

## Regulation of PLCε

### Membrane association

PLCε is cytoplasmic under basal conditions, but has quantifiable basal activity, indicating it independently engages the membrane. At minimum, the active site in the TIM barrel must interact with the membrane for substrate binding, but other regions likely contribute. In addition to the PH domain, the N-terminal ∼300 residues and CDC25 domain likely contribute to membrane binding, as truncations of these regions decrease basal and G protein-stimulated activities (8) (Fig. 2). Although the N-terminus of PLCε1a is highly conserved (64–100% identity) across homologs (13), it does not share sequence homology to other proteins. Apart from the CDC25 domain, it is also not clear whether the N-terminus of the lipase is structured. While AlphaFold3 does not predict any secondary structure (34), algorithms that predict intrinsically disordered regions (35, 36) instead flag these residues as ordered, albeit with varying confidence.

The guanine nucleotide exchange factor (GEF) activity was localized to the N-terminus of the lipase using N-terminal truncations that removed the first ∼1,200 residues of the protein (37, 38). Comparison of the PLCε N-terminus to other GEFs identified the Ras GEF Son-of-Sevenless as the closest homolog, and their CDC25 domains share 22% identity (38). AlphaFold3 models predict the CDC25 domain spans residues 510 to 780, with additional structured regions in the ∼50 residues preceding the domain, though with lower confidence (34). Intriguingly, these models all predict an extensive interface between the CDC25 and PH domains (7, 34). Like the PH domain, the CDC25 domain features a surface with conserved, solvent-exposed basic and hydrophobic residues. In the CDC25/PH module, these surfaces are continuous and would form a nonspecific membrane interaction site in the same plane as the lipase active site (Fig. 2). Mutations at the predicted CDC25/PH interface decreased activity, but whether this is due to solely to disruption of the interdomain interface could not be established. Mutation of basic and hydrophobic residues on the potential membrane binding surface of the CDC25/PH module did decrease both basal and G protein-stimulated activities (7). Further studies are needed to confirm these decreases are due in fact to defects in membrane association.

### Autoinhibition

PLCε is autoinhibited by the X–Y linker, as its deletion increases basal activity ∼10-fold in cells, most likely by exposing the active site (29). While the X–Y linker is largely unconserved in sequence and length, PLCε, PLCβ, and PLCδ enzymes contain a highly conserved 10 to 15 residue acidic stretch (29). In interfacial activation, the unfavorable electrostatic interactions between this acidic stretch and the negatively charged inner leaflet of the plasma membrane displace the linker and expose the active site. The α_X–Y_ helix at the N-terminus of the PLCε X–Y linker may also participate by interacting with the membrane, given that amphipathic helices are well-established membrane binding elements (39) and deletion of the α_X–Y_ helix significantly decreased basal activity in cells (6). Alternatively, the α_X–Y_ helix may be more important for activation at the perinuclear membrane, where interfacial activation would be less efficient due to the decreased charge of its cytoplasmic leaflet (40, 41). Future studies are needed to validate the mechanism of interfacial activation, how the PLCε active site is exposed at the perinuclear membrane, and the role of the α_X–Y_ helix.

The Y-box may also contribute to membrane association, given its location on the TIM barrel and proximity to the X–Y linker. While this insertion is disordered in all structures of PLCε to date (6, 7, 17), it may become ordered at the membrane. This may explain why deletion of the Y-box profoundly decreases lipase activity (10, 17).

## Activation of PLCε by G proteins

### Regulation by Rap1A

**Figure 4:**
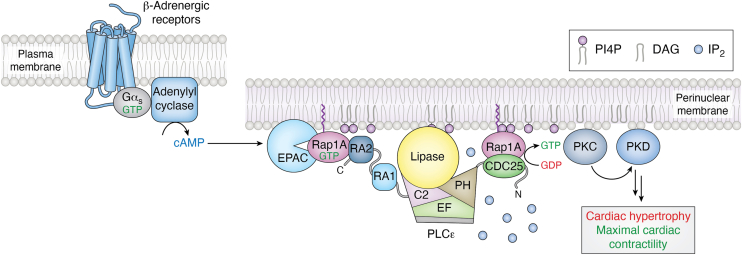
**Rap1A activates PLCε at the perinuclear membrane**. Following β-adrenergic receptor stimulation, Gα_s_ activates adenylyl cyclase to produce cAMP. The cAMP activates Epac, a GEF for Rap1A. Rap1A•GTP in turn binds to the PLCε RA2 domain, translocating and activating the lipase at the perinuclear membrane. The complex is stabilized at the membrane through interactions between the PLCε Ras association domains and mAKAP (not shown). PLCε hydrolyzes PI4P, generating IP_2_ and DAG, the latter of which activates PKC and PKD. Under normal conditions, this pathway maximizes cardiac contractility. Sustained activation is proposed to be driven by a feed-forward loop in which the GEF activity of PLCε creates a local pool of activated Rap1A, which in turn binds the lipase to stimulate PI4P hydrolysis. Ultimately, this leads to increased transcription of genes that drive cardiac hypertrophy. PI4P is shown in *purple* and IP_2_ is shown in *light bluespheres*. PLCε domains are colored as in Figure 1*A*. DAG, diacylglycerol; IP_2_, inositol-1,4-phosphate, GEF, guanine nucleotide exchange factor; PKC, protein kinase C; PKD, protein kinase D; PLCε, phospholipase Cε.

Rap1A is the best characterized activator of PLCε. Stimulation of β-adrenergic receptors leads to activation of adenylyl cyclase, and the resulting increase in cAMP activates Exchange Protein Activated by cAMP (Epac) (42). Epac is a GEF for Rap1A, and once activated, Rap1A•GTP binds the RA2 domain of PLCε, translocating and activating the lipase at the perinuclear membrane or Golgi (12). In the cardiovascular system, the Rap1A–PLCε complex is maintained at this location by binding to the scaffold mAKAP via the RA domains (33). At the perinuclear membranes, PLCε hydrolyzes PI4P, producing IP_2_ and DAG (Figs. 3*B* and 4).

The PLCε CDC25 domain is thought to be essential for sustained activation of the lipase by Rap1A. This domain is specific for Rap1A, and does not activate even closely related small GTPases, such as Rap2 (60% identity). Comparing the predicted structure of the CDC25 domain to experimentally determined structures of Rap1 GEFs including Epac2 (RAPGEF4) (PDB: 3CF6) and C3G (RAPGEF1), reveal a conserved mode of GTPase binding that precisely engages switch II of Rap1A (43, 44, 45). The PLCε GEF activity produces a local pool of Rap1A•GTP, which then binds to the PLCε RA2 domain to increase lipase activity, thus sustaining PI4P hydrolysis (46).

Rap1A•GTP binds to the RA2 domain, but other regions of PLCε, namely the PH domain, are essential for activation to occur (16). Small angle X-ray scattering studies comparing Rap1A–PLCε PH-C and another N-terminal truncation, EF3-C, showed that binding of the G protein induced long-range conformational changes, indicating the mechanism involves an allosteric component (47). How Rap1A binding to the flexibly tethered RA2 domain induces conformational changes that increase PLCε activity is not clear. One possible mechanism is that the Rap1A-bound RA2 domain interacts with other regions in PLCε, such as the PH domain. Alternatively, there may be a second, lower affinity binding site on PLCε for Rap1A. Rap1A binding, either to a single site or multiple sites, may serve to stabilize the CDC25 and RA2 domains in close proximity to one another. This would facilitate the transfer of newly activated Rap1A between the domains and promote feed-forward activation. Furthermore, the GEF activity of PLCε may be further enhanced by binding of Rap1A•GTP, as has been reported for allosteric activation of SOS by Ras•GTP (47, 48).

### Regulation by RhoA

**Figure 5:**
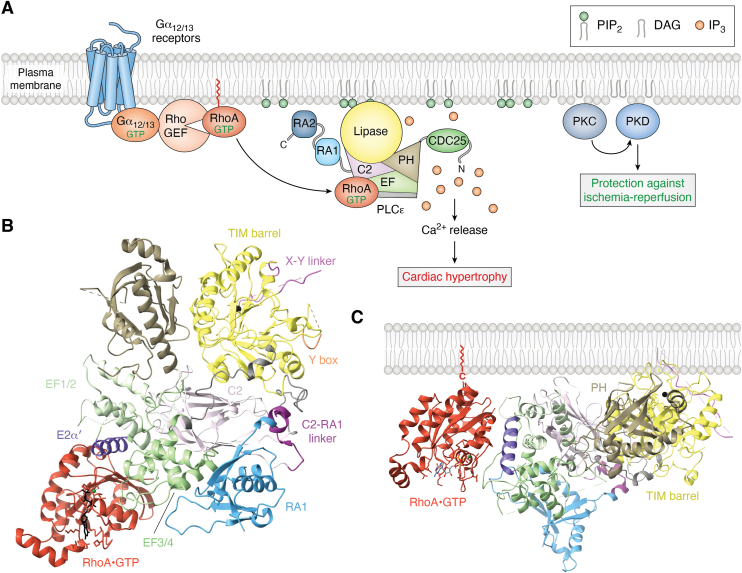
**RhoA activates PLCε at the plasma membrane**. *A*, Stimulation of G_12/13_-coupled receptors results in the activation of RhoGEFs. Once activated, RhoA•GTP binds PLCε *via* the E2α′ helix in the EF hands and increases PIP_2_ hydrolysis at the plasma membrane through a mechanism involving membrane localization and allostery. The DAG produced by PIP_2_ cleavage activates PKC, which phosphorylates and activates PKD. In response to ischemia-reperfusion injury, this pathway prevents mitochondrial lysis and subsequent cardiomyocyte death. However, increased activation of this pathway results in elevated Ca^2+^, and ultimately cardiac hypertrophy and heart failure. PIP_2_ is shown in *green*, and IP_3_ in *orangespheres*. *B*, Cryo-EM reconstruction of PLCε PH-C (residues 837–2282) bound to RhoA•GTP (PDB ID: 9AX5). The switch regions of RhoA•GTP bind to the E2α′ helix (17). *C*, When RhoA•GTP binds to the EF hands, its prenylated C-terminus and the PLCε PH and TIM barrel domains and PH domain are aligned in the same plane and can simultaneously interact with the membrane. PLCε is colored as in Figure 1*A*, the E2α′ helix in *dark purple*, and RhoA•GTP in *burnt orange*. DAG, diacylglycerol; GEF, guanine nucleotide exchange factor; IP_3_, inositol-1,4,5-triphosphate; PIP_2_, phosphatidylinositol-4,5-bisphosphate; PKC, protein kinase C; PKD, protein kinase D; PLCε, phospholipase Cε.

RhoA-dependent activation occurs in response to stimulation of G_12/13_-coupled receptors, including those for thrombin, lysophosphatidic acid, and sphingosine-1-phosphate. Gα_12/13_•GTP binds and stimulates RhoGEFs, which catalyze the exchange of GDP for GTP on Rho GTPases (14, 49, 50) (Fig. 5*A*). Of the Rho subfamily, RhoA is the most robust activator of the lipase, increasing PLCε-dependent PIP_2_ hydrolysis up to ∼10-fold at the plasma membrane (15, 17, 51, 52, 53).

The first mechanistic studies of RhoA-mediated activation of PLCε focused on the Y-box, as its deletion eliminated RhoA-dependent activation, but not binding (14, 15). The cryo-EM reconstruction of a RhoA•GTP–PLCε PH-C complex (PDB ID: 9AX5) revealed that RhoA•GTP bound exclusively to the EF hands, on the opposite side of the TIM barrel from the Y-box (17). The switch regions of RhoA bind directly to the E2α′ helix within the EF hands (Fig. 5*B*). This not only confirms why the lipase only interacts with the activated G protein, but also why PLCε variants lacking the EF1/2 subdomain were not activated by RhoA in cells (17). The orientation of RhoA•GTP in the complex is compatible with membrane binding, as its prenylated C-tail lies in the same plane as the active site in the TIM barrel (Fig. 5*C*). The mechanism of activation requires both membrane association and allostery, as soluble, active mutants of RhoA only partially activate the lipase (17). How RhoA binding to the EF hands is relayed to the active site is not known. One possibility is that RhoA•GTP binding may induce intramolecular conformational changes within the TIM barrel that facilitate displacement of the X–Y linker from the active site.

The PLCε E2α′ helix is only necessary for RhoA-dependent activation. Deletion of the helix had no impact on activation by Rap1A or the Gβγ heterodimer. Conversely, insertion of E2α′ into PLCβ3, which is not regulated by RhoA, was sufficient to confer some sensitivity to the GTPase (17). In addition, RhoA and Ras GTPases have been shown to simultaneously activate PLCε (15). Thus, it seems there are at least two independent mechanisms for G protein-mediated activation of the lipase.

### Regulation by Ras GTPases

**Figure 6:**
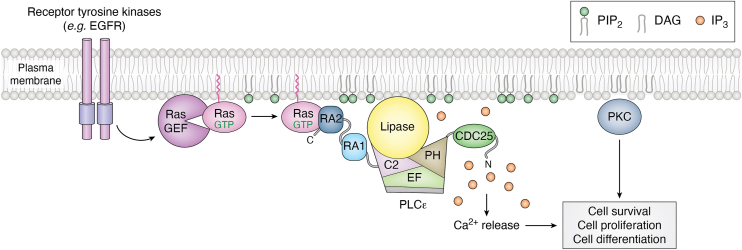
**Ras activates PLCε at the plasma membrane**. Ras GTPases are activated in response to receptor tyrosine kinase stimulation. Ras•GTP binds to PLCε *via* its RA2 domain, and activates the lipase, at least in part, through localization to the plasma membrane. Ras-dependent activation increases PIP_2_ hydrolysis, promoting Ca^2+^ release and PKC activation, which initiate pathways necessary for cell survival, proliferation, and differentiation. PIP_2_ is shown in *green* and IP_3_ in *spheresorange*. PLCε is colored as in Figure 1*A*. PLCε, phospholipase Cε; PKC, protein kinase C; IP_3_, inositol-1,4,5-triphosphate; PIP_2_, phosphatidylinositol-4,5-bisphosphate.

PLCε was initially identified as a putative Ras effector in a yeast two hybrid screen in *C*. *elegans* (54). Subsequent studies showed that, following stimulation of receptor tyrosine kinases, specifically growth factor receptors, Ras bound directly to the C-terminal RA2 domain of PLCε, translocating the lipase to the plasma membrane for PIP_2_ hydrolysis (5, 9, 11, 15) (Fig. 6). The crystal structure of H-Ras^G12V^•GTP bound to the isolated RA2 domain (PDB ID: 2C5L) confirmed that PLCε interacts only with the activated G protein *via* its switch regions (9). How Ras binding increases lipase activity is not yet clear. Because Ras is prenylated, it was initially proposed to activate PLCε by membrane translocation, and once at the membrane, interfacial activation would expose the active site (9, 11). However, a membrane-tethered PLCε was further activated by Ras (9), suggesting an allosteric component. There may also be more than one Ras binding site on the lipase, as a membrane-tethered PLCε variant lacking the RA2 domain was still activated by Ras in response to stimulation of the epidermal growth factor receptor (9).

While a comprehensive investigation into the domains necessary for Ras activation has yet to be carried out, the N-terminus and CDC25 domains do not appear to be required. Indeed, the PLCε CDC25 domain has no GEF activity towards any Ras isoform tested (37). One possible mechanism for Ras-dependent activation is that the RA2 domain is required for initial binding to Ras at the plasma membrane. Once at the membrane, interactions between Ras, the RA2 domain, and/or other regions of the lipase promote interfacial activation and optimize the orientation of the PLCε active site for PIP_2_ hydrolysis.

### Regulation by the Gβγ heterodimer

Once a PH domain had been predicted in PLCε, it was hypothesized the lipase may be regulated by the Gβγ heterodimer (8, 13). Gβγ is well-known to bind PH domains in other signaling enzymes, including GPCR kinase 2 (GRK2) (13, 55) and it increases the activity of some PLCβ isoforms (56, 57) and the PLCη (58) subfamily. In cell-based assays, co-transfection with Gβγ and PLCε resulted in increased lipase activity. This increase was presumed to be due to direct binding, as co-transfection of other Gβγ binding partners, such as Gα_i_•GDP or the PH domain of GRK2, inhibited activity (13, 59). This was later validated *in vitro*, as purified Gβγ activated PLCε PH-C in a liposome-based assay. Efforts to map the Gβγ binding site on PLCε relied on N- and C-terminal truncations of the lipase, and the N-terminus, CDC25 domain, and RA2 domain were found to be necessary for maximum activation (59). Once again, activation is unlikely to be strictly dependent on membrane localization, as the activity of a membrane-tethered PLCε was further increased by Gβγ (59). At present, two models have been proposed for Gβγ binding to the lipase; one in which one Gβγ molecule binds to the N-terminus of the lipase and a second Gβγ binds to the RA2 domain, or one Gβγ binds to a single binding site formed by N-terminus, CDC25 and RA2 domains (59). This question, as well as the mechanism of activation, require further study.

**Figure 7:**
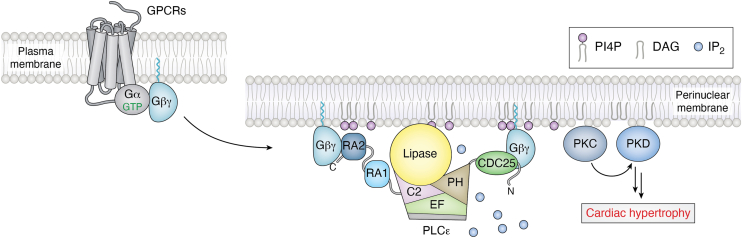
**Gβγ activates PLCε at the perinuclear membrane**. Gβγ is released following stimulation of the endothelin-1 receptor and binds to PLCε. The Gβγ heterodimer may interact with a single binding site, formed by the N-terminus, CDC25, and RA2 domains, or 2 Gβγ molecules may bind, one at the N-terminus and one at the C-terminus, as shown. Gβγ activates PLCε at the perinuclear membrane for PI4P hydrolysis. This results in PKC- and PKD-dependent phosphorylation and increased expression of cardiac hypertrophy genes. PI4P is shown in *purple*, and IP_2_ in *light blue* spheres. PLCε is colored as in Figure 1*A*. CDC25, cell division and cycle 25; DAG, diacylglycerol; IP_3_, inositol-1,4,5-triphosphate; PIP_2_, phosphatidylinositol-4,5-bisphosphate; PKC, protein kinase C; PKD, protein kinase D; PLCε, phospholipase Cε.

Gβγ-dependent regulation of PLCε in the cardiovascular system was identified indirectly. Stimulation of the endothelin-1 receptor (ET1R) caused translocation of PLCε to the perinuclear membrane. However, because ET1R couples to both G_12/13_ and G_q_ heterotrimers, the connection to PLCε was not immediately apparent (3). Subsequent studies showed that ET1R regulates PLCε *via* Gβγ, as inhibition of Gβγ signaling blocked PI4P hydrolysis at the perinuclear membrane. Indeed, inhibiting Gβγ-dependent activation of PLCε prevented the development of cardiovascular disease (60), indicating this pathway also likely increases the expression of hypertrophic genes (Fig. 7).

## Physiological roles of PLCε

### Cardiovascular disease

PLCε plays critical roles in the cardiovascular system. In cardiomyocytes, PLCε canonically hydrolyzes PIP_2_ at the plasma membrane, generating IP_3_ and DAG. IP_3_ binds to its receptors on the sarcoplasmic/endoplasmic reticuli, triggering Ca^2+^ release from intracellular stores. The increased Ca^2+^, in combination with DAG, activates PKC isoforms, initiating pathways that are required for sustaining maximal cardiac contractility and normal cardiac function (42, 61). PLCε is essential for maximum Ca^2+^-induced Ca^2+^ release (CICR) in cardiac myocytes following stimulation of β-adrenergic receptors. In *PLCE* knockout mice, this pathway is disrupted and results in reduced Ca^2+^ release, decreased pumping ability, and reduced adaptation to physiological stresses (61, 62). Elevated PLCε expression has also been reported in failing human hearts, likely due to compensatory responses aimed at restoring normal cardiac function (61, 63). However, overexpression, dysregulation, or sustained activation of PLCε through this pathway can lead to cardiac hypertrophy and heart failure, as it results in the upregulation of genes that promote cardiac hypertrophy. This drives maladaptive remodeling, including fibrosis (3, 33, 42, 49, 61).

PLCε is also activated downstream of G_12/13_-coupled receptors, which leads to its activation at the plasma membrane. In the aftermath of ischemia/reperfusion injuries, this pathway is initially cardioprotective. PIP_2_ hydrolysis by PLCε results in PKC activation, which in turn activates protein kinase D and prevents translocation of a pro-apoptotic complex to the mitochondria (64). This protects the mitochondria and prevents cardiomyocyte death (64, 65). However, sustained activation of PLCε through this pathway also results in elevated Ca^2+^, leading to activation of Ca^2+^/calmodulin-dependent protein kinase II, and ultimately upregulation of genes that promote cardiac hypertrophy (66, 67).

### Insulin secretion and nephropathy

In the pancreas, PLCε is expressed in islets of Langerhans where it is required for CICR in β-cells following stimulation of glucagon-like peptide-1 receptors (68). This regulates insulin secretion after plasma membrane depolarization, as the IP_3_ from PIP_2_ hydrolysis promotes CICR at the ER, and DAG and Ca^2+^ activate PKC to initiate the exocytosis of insulin-containing vesicles (69, 70, 71, 72). Dysregulation of PLCε decreases the Ca^2+^ mobilization required for glucose-stimulated insulin secretion and leads to insulin resistance, a hallmark of Type 2 diabetes (68).

In the kidneys, PLCε is expressed in the podocytes and glomeruli during early stages of development (73). PLCε is required for migration, proliferation, and proper maturation of podocytes (74) and glomeruli (73). Mutations in the lipase (73, 75) or its interaction partners (76) can lead to various types of nephrotic syndrome, including steroid resistant, diabetic, and hypertensive (68, 75, 77, 78, 79). Mutations in PLCε have been detected in familial nephrotic syndrome and are widespread throughout the lipase (56). Mutations within EF3/4 and the TIM barrel have been computationally characterized, and are predicted to alter lipase stability, substrate binding, and/or catalytic activity (77). These results remain to be experimentally validated, especially in the context of nephrotic syndrome.

### Cancer

Activation of PLCε leads to changes in cell proliferation (80), apoptosis (81), and inflammation (46, 82, 83, 84), all of which can promote oncogenesis and progression. PLCε has been linked to several cancers (85), among them skin (86), lung (87), gastrointestinal/esophageal (88, 89, 90, 91, 92, 93, 94), colorectal (95, 96, 97), prostate (98, 99, 100), and bladder (80, 81, 101, 102, 103). Genome-wide association studies have revealed several mutations in the coding region of *PLCE* that increase the risk of developing cancer. Many of these mutations are located within the TIM barrel or C2 domain, with some reportedly increasing lipase activity (104). Whether the mutations change activity by altering the structure, disrupting autoinhibitory interactions, and/or binding of regulatory proteins has yet to be determined. The role of PLCε in cancer is complex, as it is reported to be a tumor suppressor in some cancers (83, 86), and an oncogene in others (82, 86, 88).

## Conclusions and future directions

PLCε is a complex, multifunctional protein whose structure, regulation, and roles in health and disease are now beginning to be understood. Cryo-EM reconstructions of larger fragments of PLCε have helped define the structures of basal and G protein-bound conformations of the lipase. These studies experimentally confirmed two PLCε N-terminal domains, identified potential membrane binding surfaces, and revealed the architecture of the EF hands to be the key in RhoA binding and activation. However, numerous questions remain, such as the structure and function of the PLCε N-terminal ∼300 residues. Its conservation across eukaryotes speaks to its importance, but how this region contributes to PLCε activity is not clear. In addition, the AlphaFold3 predictions regarding the CDC25 domain remain to be experimentally validated. This includes the molecular basis for its specificity for Rap1A and whether the interface between the CDC25 and PH domains is functionally relevant. To capture these regions, and ultimately the lipase in its active state, structural studies of the full-length protein on a model membrane will be required.

Maximum PLCε activity is only observed when the lipase and a G protein interact at a membrane. All known activators of PLCε are prenylated and may recruit the lipase to the membrane and/or increase its affinity for the surface. At minimum, G protein binding to PLCε likely contributes to optimizing the orientation of the active site at the membrane for interfacial activation and PIP hydrolysis. However, this is insufficient for maximum activation. This is best illustrated in the case of RhoA•GTP-dependent activation, where soluble mutants of the G protein were sufficient to partially actiate the lipase (17). Ras and Gβγ also further increased the activity of membrane-anchored PLCε variants (9, 59). The two structures of G protein–PLCε complexes also indicate there is more than one way to activate PLCε (9, 17). By binding structurally distinct sites, Ras and RhoA additively activate PLCε (15) at the plasma membrane for PIP_2_ hydrolysis. Rap1A and Gβγ both activate PLCε at the perinuclear membrane, but it is less clear whether additive, or even synergistic activation is possible, especially as the stoichiometries of these activated G protein–PLCε complexes remain to be determined. Such combinatorial regulation would allow fine-tuning of PLCε activity.

The temporal and signal termination components of PLCε regulation have yet to be investigated. Relative to other PLC enzymes, which are rapidly and robustly activated in response to receptor stimulation (105), PLCε activation is slower and sustained (46, 106). The GEF activity of PLCε provides an explanation for this in the case of Rap1A, but it is unclear how this is achieved in other activation pathways. How G protein-dependent activation of PLCε is terminated is also not known. In the case of PLCβ, it has GTPase activating protein (GAP) activity, and so stimulates GTP hydrolysis on Gα_q_ (20, 27). Gα_q_•GDP reassociates with Gβγ and the signal terminates. Thus far, there has been no evidence of GAP activity in PLCε, but the possibility cannot be excluded. Alternatively, there may be GAPs that associate with PLCε at the membrane to facilitate signal termination.

As a key player in GPCR and receptor tyrosine kinase signaling at two distinct membrane surfaces, it is unsurprising that PLCε activity is important. Most studies have investigated the roles of PLCε in the cardiovascular system, where it is required for maximum contractility (3, 12, 33, 61). Dysregulation of its activity and/or expression results in cardiac hypertrophy and heart failure, likely driven by the ability of PLCε and the Rap1A GTPase to establish a feed-forward loop that changes gene expression. More recent studies show that PLCε plays essential roles in other CICR pathways, including insulin secretion, kidney development, and nephrotic syndrome (68, 72, 73, 76). PLCε is linked to cancer, with mutations in the coding region linked most clearly linked to gastric and esophageal cancers (80, 81, 88, 104). Its role in other tumors is highly context-dependent, as the lipase is reported to have tumor suppressor and oncogenic behavior (82, 83, 86, 88). Identifying mechanisms of regulation will be essential for a comprehensive understanding of PLCε activity and its dysregulation, in health and disease.

## Conflict of interest

The authors declare that they have no conflicts of interest
